# Delving into the lifestyle of Sundarban Wetland resident, biofilm producing, halotolerant *Salinicoccus roseus*: a comparative genomics-based intervention

**DOI:** 10.1186/s12864-023-09764-w

**Published:** 2023-11-13

**Authors:** Bhramar Dutta, Urmi Halder, Annapurna Chitikineni, Rajeev K. Varshney, Rajib Bandopadhyay

**Affiliations:** 1 Department of Botany, Microbiology Section, The University of Burdwan, Burdwan, West Bengal-713104 India; 2 Center of Excellence in Genomics and Systems Biology, International Crops Research Institute for the Semi-Arid Tropics (ICRISAT), Hyderabad, India; 3 State Agricultural Biotechnology Centre, Centre for Crop and Food Innovation, Murdoch University, Murdoch, 6500 Australia

**Keywords:** *Salinicoccus roseus*, Environmental adaptation, Biofilm, Genome sequencing, Comparative genomics

## Abstract

**Background:**

Microbial community played an essential role in ecosystem processes, be it mangrove wetland or other intertidal ecologies. Several enzymatic activities like hydrolases are effective ecological indicators of soil microbial function. So far, little is known on halophilic bacterial contribution and function on a genomic viewpoint of Indian Sundarban Wetland. Considering the above mentioned issues, the aims of this study was to understand the life style, metabolic functionalities and genomic features of the isolated bacterium, *Salinicoccus roseus* strain RF1H. A comparative genome-based study of *S*. *roseus* has not been reported yet. Henceforth, we have considered the inclusion of the intra-species genome comparison of *S*. *roseus* to gain insight into the high degree of variation in the genome of strain RF1H among others.

**Results:**

*Salinicoccus roseus* strain RF1H is a pink-red pigmented, Gram-positive and non-motile cocci. The bacterium exhibited high salt tolerance (up to 15% NaCl), antibiotic resistance, biofilm formation and secretion of extracellular hydrolytic enzymes. The circular genome was approximately 2.62978 Mb in size, encoding 574 predicted genes with GC content 49.5%. Presence of genomic elements (prophages, transposable elements, CRISPR-Cas system) represented bacterial virulence and multidrug-resistance. Furthermore, genes associated with salt tolerance, temperature adaptation and DNA repair system were distributed in 17 genomic islands. Genes related to hydrocarbon degradation manifested metabolic capability of the bacterium for potential biotechnological applications. A comparative pangenome analysis revealed two-component response regulator, modified C4-dicarboxylate transport system and osmotic stress regulated ATP-binding proteins. Presence of genes encoding arginine decarboxylase (ADC) enzyme being involved in biofilm formation was reported from the genome. In silico study revealed the protein is thermostable and made up with ~ 415 amino acids, and hydrophilic in nature. Three motifs appeared to be evolutionary conserved in all *Salinicoccus* sequences.

**Conclusion:**

The first report of whole genome analysis of *Salinicoccus roseus* strain RF1H provided information of metabolic functionalities, biofilm formation, resistance mechanism and adaptation strategies to thrive in climate-change induced vulnerable spot like Sundarban. Comparative genome analysis highlighted the unique genome content that contributed the strain’s adaptability. The biomolecules produced during metabolism are important sources of compounds with potential beneficial applications in pharmaceuticals.

**Supplementary Information:**

The online version contains supplementary material available at 10.1186/s12864-023-09764-w.

## Background

The Ganges River delta, also named as Sundarbans, is the world’s largest mangrove ecosystem, spreaded across India (~ 40%) and Bangladesh (~ 60%). The Indian side of Sundarban designated as Wetland of International Importance (Ramsar Site no. 2370) [1], due to its diverse ecological communities, unique species composition and rich biodiversity. Albeit of its unique biodiversity and ecological importance, currently the Sundarbans is threatened by both anthropogenic pressures and climate change [2]. Microbial community played important role in carbon sequestration, nutrient cycling, and decomposition in mangrove wetland [3]. However, research done so far on soil microbial diversity of Sundarban were based on 16S rRNA gene sequencing, comprehensive analysis of entire genomic DNA sequencing of halophilic bacteria remained pretty limited [4, 5]. Understanding the nutrient utilization and spatial heterogeneity that might prompt the microorganism to shape their genome is crucial. The genomic contents and metabolic versatility define a microorganism’s ability to feed on available nutrients, for example, nitrogen is a relevant driver of genome size [6]. Characterizing wetland resident bacteria that played key role in nutrient cycling and predicting their responses to changing climate is very important. These investigations represent an important step towards effective management of intertidal ecology in a genomic perspective.

The genus *Salinicoccus* belongs to the family Staphylococcaceae. Species of this genus was first introduced from Alicante, Spain [7] and was characterized as Gram-positive, aerobic, non-motile, spherical-shaped bacteria. A group of *Salinicoccus* exhibited moderately halophile (3–15% NaCl tolerance) and intense pink pigmentation, which are mostly dwell in saline environments [8, 9]. In total, three sampling points were considered for the whole study *viz*., Riverfront I, Riverfront II and Pneumatophore inhabiting areas of Indian Sundarban. A halophilic strain of *Salinicoccus roseus* RF1H was isolated from the riverfront area of Sundarban [10].

So far, complete genome of only five strains of *Salinicoccus roseus* are available in NCBI *viz.*, strain BU-1 [11], strain W12 [12], strain CCM 3516 [13], strain DSM 5351^ T^ and strain MOSEL-ME25 (as on 17^th^ May, 2022), and all are isolated from diverse habitats. The genomes of *S*. *roseus* have been analyzed in several studies and functional proteins have been characterized [12]. However, a comparative genome-based study delineating functional systems and genome evolution of *S*. *roseus* is still not fully explored. Therefore, a detailed analyses of *S*. *roseus* genome obviously needed to understand physiological mechanisms involved in salt tolerance and potential metabolic activities involved in this species. Moreover, comparative genomics allowed us to identify genes which are present in any particular genome, also which ones are absent. In addition, it advanced our understanding of adaptive mechanism of the strain RF1H, which is undoubtedly state-of-the-art.

The biofilm production by *S*. *roseus* is growth-associated and defense mechanism against antibacterial agents. Like other biofilm forming bacteria, halophiles aggregate in extracellular polymeric matrix for surface adhesion and develop resistance to high salt damages [14]. Polyamine spermidine, a polycationic aliphatic hydrocarbon regulates biofilm formation in divergent bacterial taxa [15]. Arginine decarboxylases (ADC) (EC 4.1.1.19) are important enzymes for polyamine biosynthesis in bacteria. However, the protein structure and phylogeny of ADC remains largely unknown. Here, we used an in silico approach to understand the structural and functional properties of ADC which elucidated their role in biofilm formation.

The increasing interest in the genus *Salinicoccus* for wide range of biotechnological applications like, industrial enzymes, bioremediation of toxic pollutants, development of colors from natural pigments, biofuel and pharmaceutical industries [16, 17], *S*. *roseus* demands substantial attention as a promising candidate for bio-based products. The objective of this study is to establish environmental adaptability, phylogenomics, and metabolic toolsets of *S*. *roseus* strain RF1H in a genomic perspective and how all these blueprints enable this bacterium to thrive in a hypersaline ecosystem which is extremely vulnerable to climate change.

## Results

### Morphology of the isolate

**Fig. 1:**
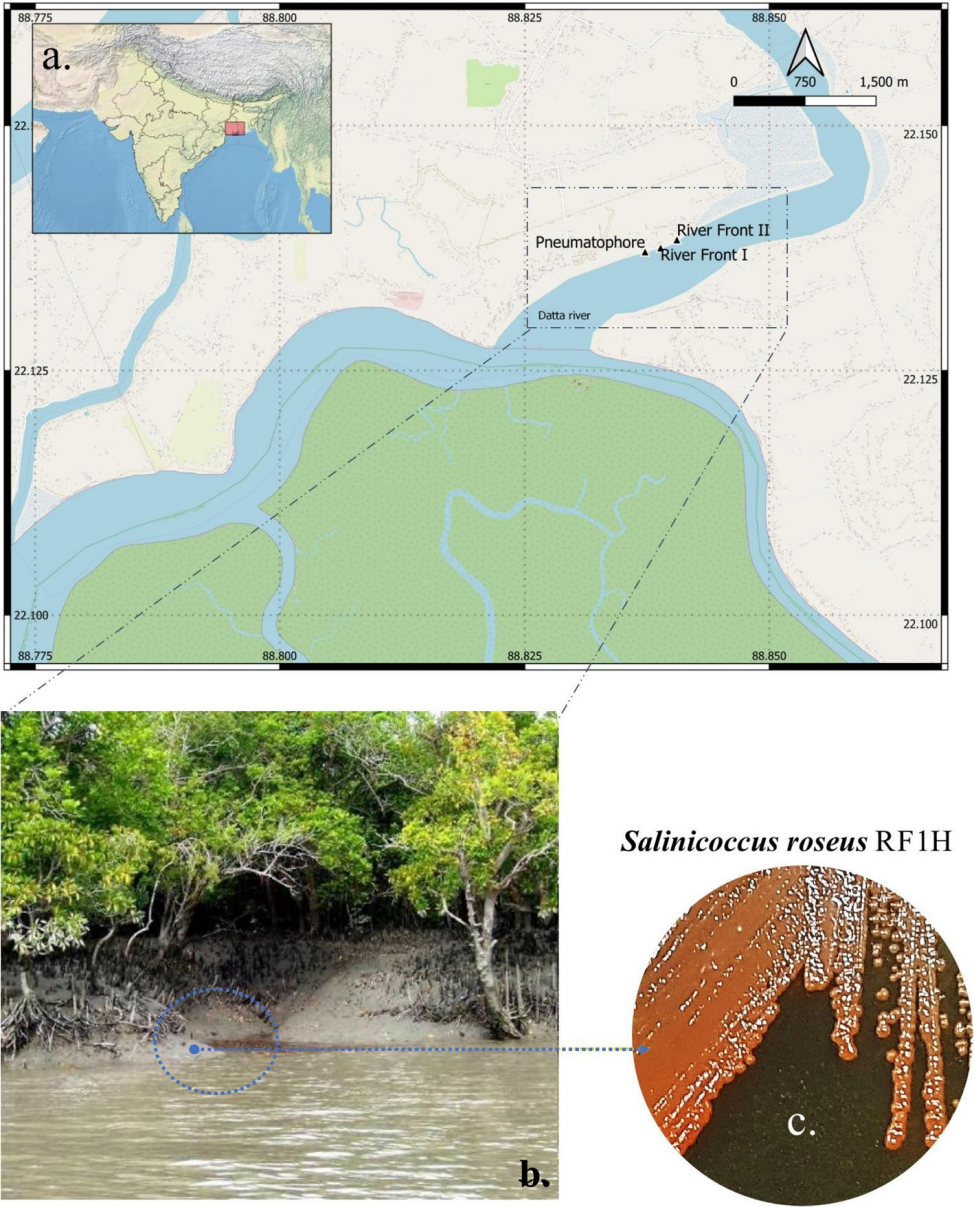
Study site of sample collection- QGIS map showing the sampling sites [background layers were obtained from Open Street Map and Natural Earth] (**a**), A section of Indian Sundarban showing the isolation site (**b**), Colony morphology of the isolated bacterial strain RF1H (**c**)

**Fig. 2:**
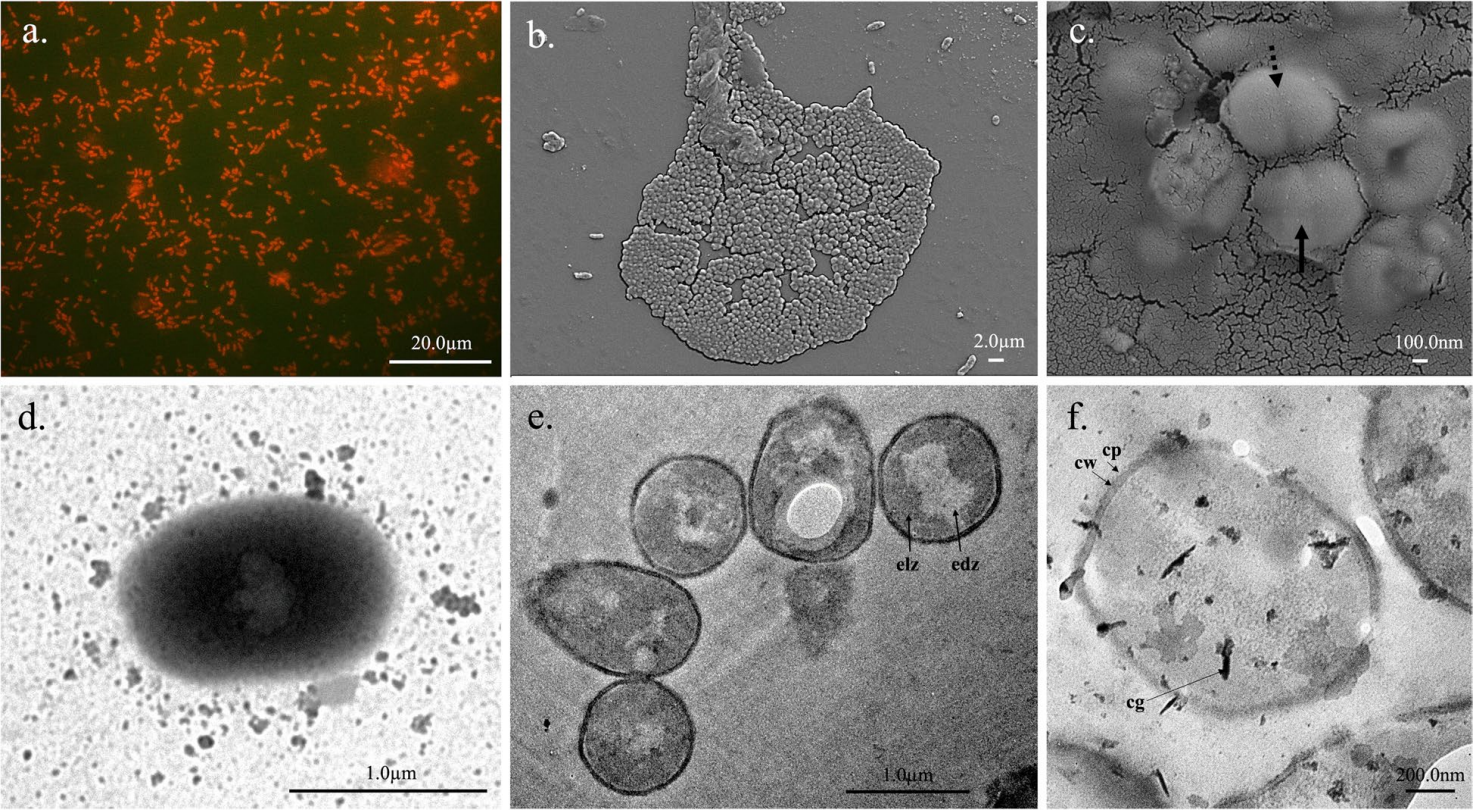
Microscopic images of the isolated bacterial strain RF1H- Fluorescence micrograph (**a**), Scanning electron micrographs (**b**, **c**), Transmission electron micrographs of RF1H cells (**d**-**f**) [electron lucid zone (elz); electron dense zone (edz); tetrad (black arrow); clumps (dotted arrow); cell wall (cw); capsule (cp) and cytoplasmic granule (cg)]

The isolation site of this study, riverfront 1 has been displayed in Fig. 1a and b. The cells of *S*. *roseus* strain RF1H formed circular, smooth, and pink-red pigmented colonies with 2.1 mm in diameter when cultured in complex halophilic agar media for 48 h at 35 °C (Fig. 1c). Fluorescence micrograph of cell showed its coccoidal shape (Fig. 2a). SEM images provided information that the cells were aggregated into biofilm matrix (Fig. 2b), more detailed structure was shown in Fig. 2c. Cells were 1.8 μm in diameter, formed tetrad or clumps. Some cells just initiated first cellular division (dotted arrow in the Fig) and some completed the second division to produce four cells in one plane (black arrow in the Fig). Further, single cell under TEM showed irregular shaped nucleoid (Fig. 2d). Cells in the group also observed (Fig. 2e). Interior of each cell contained two distinct regions—electron lucid zone (light) and electron dense zone (dark). At higher magnification, typical gram-positive cell membrane became prominent (Fig. 2f). The cell wall is surrounded by capsule. Cytoplasmic granules are found in dark colors.

### Phylogeny and genome properties

**Fig. 3:**
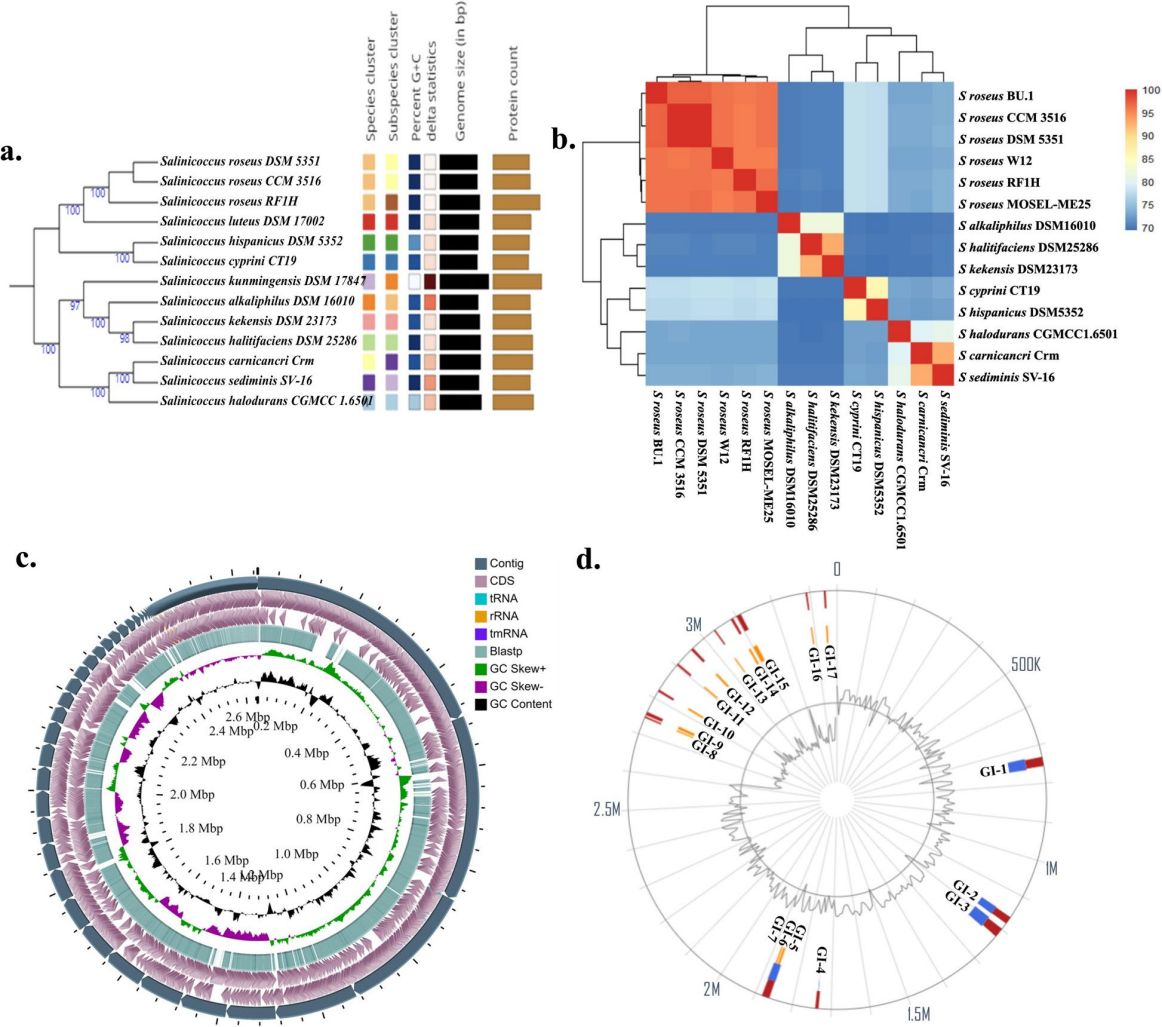
Phylogeny and genome features-TYGS genome-based phylogeny tree (**a**), ANI (%) based heat map among the close relatives of RF1H (**b**), Circular genome visualization of RF1H genome (**c**), Identified Genomic Islands of RF1H (**d**)

The phylogenetic tree constructed on TYGS depicted that the isolate RF1H formed a distinct cluster with type strain *S*. *roseus* DSM 5351^ T^ and *S*. *roseus* CCM 3516, followed by *S. luteus* DSM 17002 and the Genome BLAST Distance Phylogeny (GBDP) approach based on pairwise dDDH values between these type-strain genomes were 83.2%, 83.0%, and 60.8% respectively. Also, the difference between G + C percentages were 0.03, 0.02, and 0.39 respectively. The midpoint rooted tree was generated with average branch support of 94.9% and > 60% pseudo-bootstrap support values from 100 replications (Fig. 3a). So, RF1H belongs to the species of *Salinicoccus roseus* under the family Staphylococcaceae, order Bacillales, class Bacilli, and phylum Firmicutes. A matrix comparing the ANI (average nucleotide identity) of 14 genomes is presented in Fig. 3b, and the ANI values (%) are given in Additional file 1 Table S1. Based on ANI and whole genome similarities, *Salinicoccus roseus* and related species were initially clustered into two subgroups: Group 1 consists of *S*. *roseus* BU-1, *S*. *roseus* CCM 3516, *S*. *roseus* DSM 5351, *S*. *roseus* W12, *S*. *roseus* RF1H and *S*. *roseus* MOSEL-ME25. Group 2 comprising the species *S*. *alkaliphilus*, *S*. *halitifaciens*, *S*. *kekensis*, *S*. *cyprini*, *S*. *hispanicus*, *S*. *halodurans*, *S*. *carnicancri* and *S*. *sediminis*.

95% ANI threshold served as species level clustering delineation (Fig. 3b). ANI value < 90% is considered as wide genetic distance [18].

The genome of RF1H comprised a single 2.62978 Mb circular chromosome with 49.5% GC content (Fig. 3c). No plasmid was found. The number of coding DNA Sequences (CDSs) was 2,736. A total of 89 genes that encodes RNA of which 22 genes encode rRNAs, 63 genes encode tRNAs, and 4 genes encode ncRNA (Table 1). The number of pseudogenes were found to be 260 from the PGAP server. The genome of RF1H was further analyzed using RAST where the annotated genome subsystem coverage was 30%. The circular view of the genome represented contigs, CDS, tRNA, rRNA, tmRNA, Blast alignment with *S*. *roseus* DSM 5351^ T^, along with the GC values. Genome analysis revealed that genes responsible for salinity tolerance, such as, glycine/sarcosine methyltransferase for glycine betaine synthesis, transport system, tripartite ABC transporter as a multidrug efflux pump for lipids, peptides and natural products. The Trk and KtrB potassium transporter are highly active in hyperosmotic conditions. Membrane lipoprotein B provides resistance to osmotic shock. RAST subsystem analysis revealed the heat shock protein YciM, Hsp22.5 are associated with membrane integrity, cold shock proteins (Csps), and chaperone proteins (DnaK, GrpE) are corelated to salinity adaptation (Additional fil 1 Table S2). The genome possesses flagellar motor proteins MotA and MotB. Gram-positive bacteria *S*. *roseus* strain RF1H contained a number of late competence proteins such as ComEA, ComC, ComF. The LolB protein is distinct characteristic of Gammaproteobacteria, played crucial role in lipid anchoring of the membrane. Genes coding for the degradation of toxic compounds and other aromatic heterocyclic compounds are present in the genome. Bacterial quorum sensing (QS), a process of chemical communication conveyed to cell population for synchronization found to be regulated by *lsrACDBFGE* operon encoded autoinducing peptides (AIPs) and autoinducer-2 (AI-2). One prophage region was identified from *S*. *roseus* RF1H genome, which represented 22.7 kb of the genome size. The GC content was 51.77%. The remaining prophages were incomplete or questionable. In the encoded region, 12,575–35,341, protein similarity was found (27%) for PHAGE_Bacill_250_NC_029024(8).

**Table 1:** Main characteristics and statistics of *Salinicoccus roseus* RF1H draft genome assembly and annotation

Features	Value (CLC)	Value (NCBI)
**Genome size**	2.64767 Mb	2.62978 Mb
**GC percent**	49.6	49.5
**Number of contigs**	741	741
**Number of contigs with (PEGs)**	574	-
**Contig N50**	16726	16726
**Contig L50**	38	38
**Number of coding sequences**	3145	2736
**Genome coverage**	30.0 ×	30.0 ×
**Number of Subsystems**	266	-
**Number of RNAs**	73	89
**Completeness**	76.61%	-
**Contamination**	14.18%	-

### Genomic islands (GIs)

A total of 17 genomic islands (GIs) in RF1H strain was predicted by IslandViewer and the positional distribution of the islands is shown in Fig. 3d. Islands are cluster of genes that are evolved through a variety of processes. The first island contained a CpsD/CapB family tyrosine-protein kinase encoding autophosphorylation; transposase family protein; capsular polysaccharide biosynthesis protein and DegT/DnrJ/EryC1/StrS family aminotransferase providing antibiotic resistance (Additional file 1 Table S3). The second island demonstrated Yol-D like family that involves in SOS response; phage minor capsid protein; bacteriophage Gp15 family protein; hemolysin XhlA family protein that acts as cytotoxic to insects. SprT-like domain-containing protein, that stalls DNA replication; LLM class flavin-dependent oxidoreductase incorporated in lantibiotics production; AAA family ATPase associated with diverse cellular activities like protein homeostasis, membrane fusion, rRNA processing, molecular targeting; retron Ec67 family RNA-directed DNA polymerase/endonuclease; SDR family oxidoreductase are present in the third island. GI-3 was the largest, in terms gene content. GI-4 acquired IDEAL domain-containing protein, IS30 family transposase and competence protein ComK for horizontal transfer of genes. GI-5, GI -6 and GI-7 formed a cluster. The islands are enriched with homing endonuclease (HNH type), recombinase family protein and DUF2188 domain-containing protein for DNA damage repair. The ten shorter GIs (GI-8 to GI-17) are enriched with genes encoding for ions or carbohydrate transporters like malate:quinone oxidoreductase, Gfo/Idh/MocA family oxidoreductase, molybdate ABC transporter substrate-binding protein, cation:proton antiporter, heavy metal translocating P-type ATPase and membrane elements. Among them, GI-15 acquired some unique domain containing proteins like PAS domain, FCD domains, DUF2382 domain-containing protein.

### Intra-species genome comparison

**Fig. 4:**
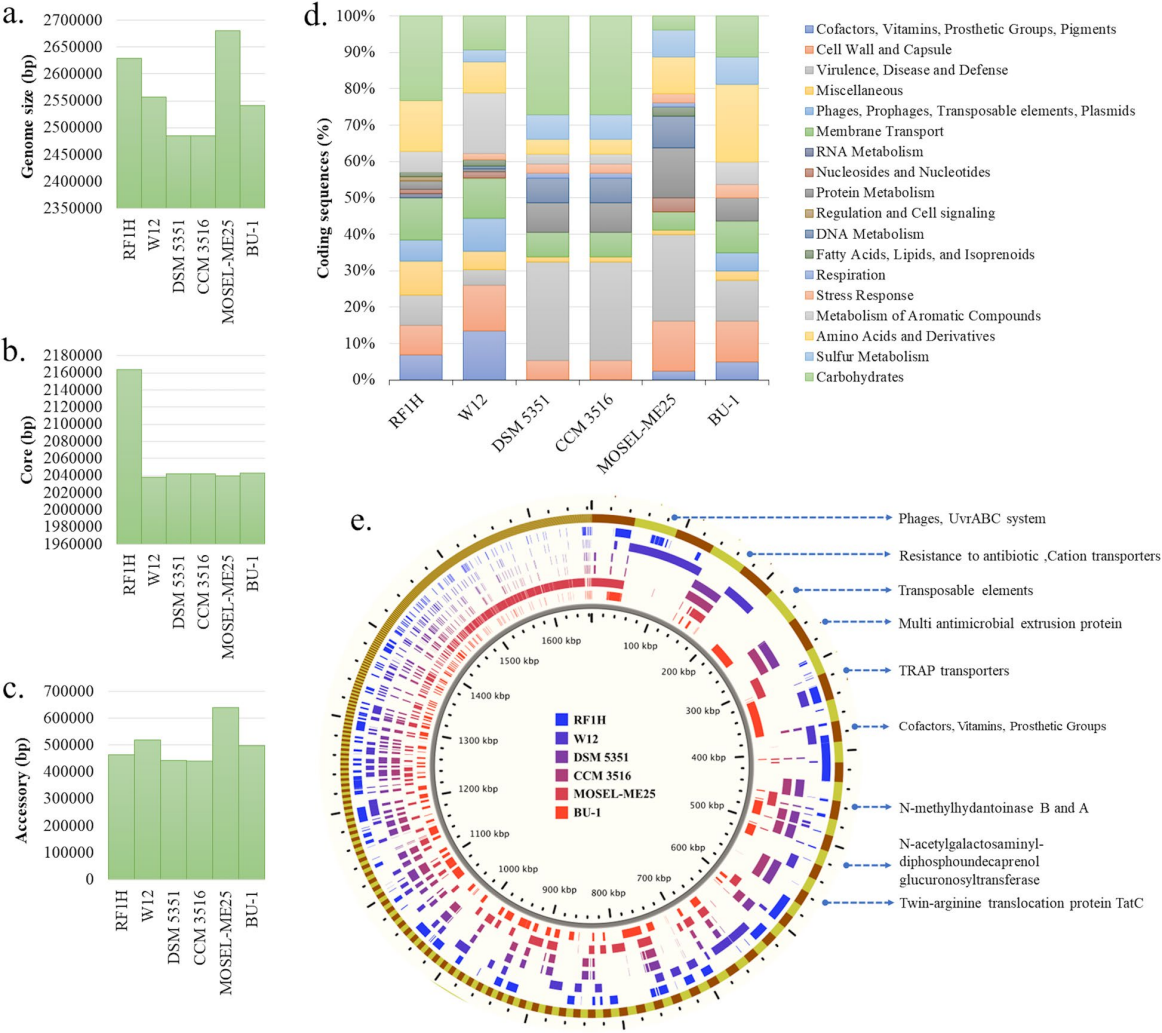
Core and accessory genome- Genome size (**a**), Core genome size (**b**), Accessory genome size (**c**), Functional annotation of accessory genomes (**d**), ClustAGE plot showing distributions of accessory genome elements of six strains of *S. roseus* (**e**) [Largest to smallest bins are represented in the outermost ring, with alternating maroon and green colors indicating the bin boundaries. Concentric inner rings showed distribution of accessory elements within each strain]

Among the available six genomes of *S*. *roseus*, strain RF1H has larger genome size of 2.62 Mb (Fig. 4a) (Additional file 1 Table S4). Core genome of strain RF1H contained maximum genes than other strains of *S*. *roseus* (Fig. 4b). Majority of the core genome fall into carbohydrate metabolism and amino acid derivatives. The core genome trends in regulation of cell signaling, cell division and membrane transport, were similar in all the strains. The accessory genomes (Fig. 4c) were enriched with secondary metabolite biosynthesis, cofactors, vitamins, pigments and aromatic carbons metabolism category (Fig. 4d) (actual values were given in Additional file 1 Table S5). Most unique genes associated with accessory genome of RF1H were rich in prophage and transposable elements, virulence and disease defense, fatty acids, and lipids content in the cell wall.

**Fig. 5:**
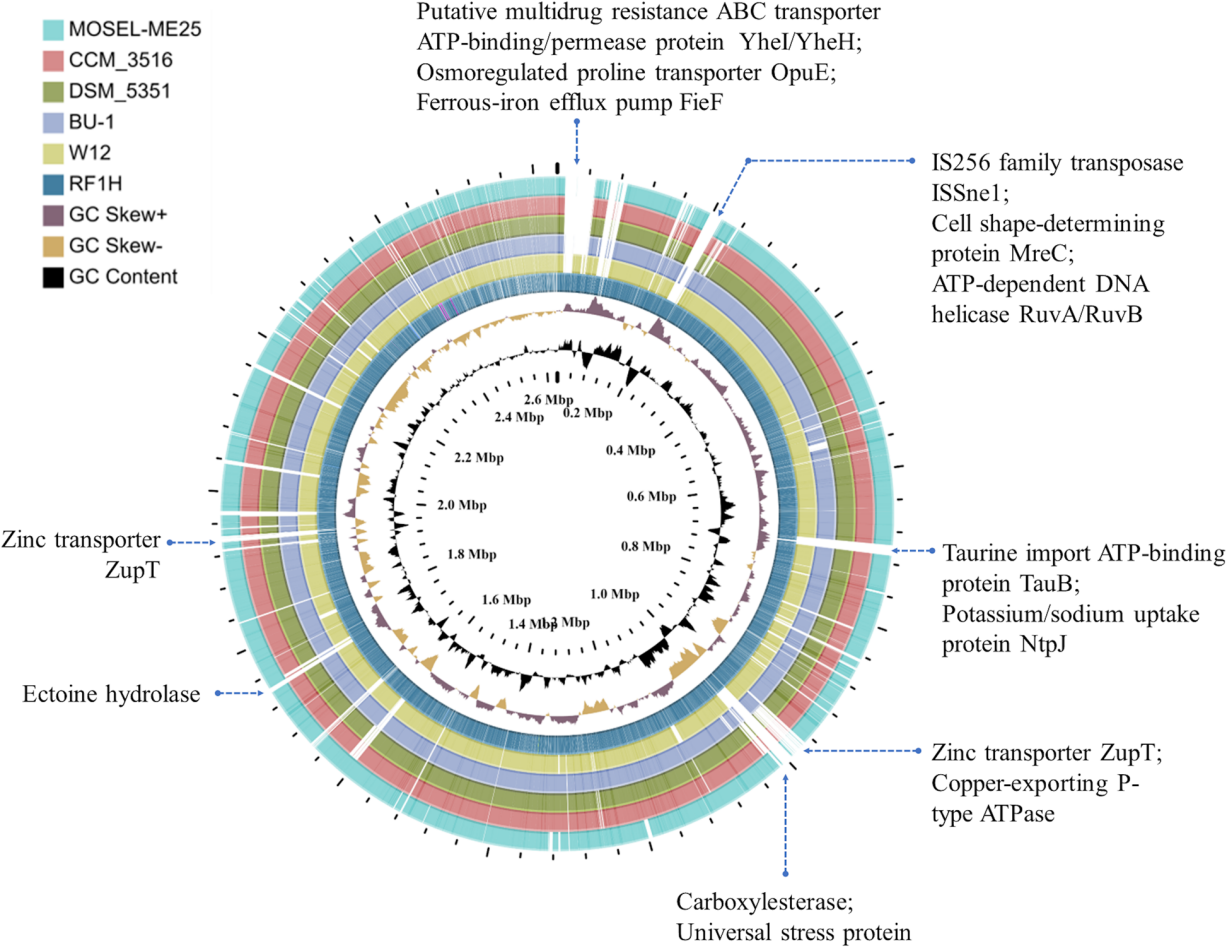
Complete circular genome visualization of all six strains of *S. roseus*

A total of 850 unique contiguous accessory genomic elements (AGEs) of 200 bp in length were identified in total input of six genomes; these were further subdivided into discrete AGE subelements of at least 100 bp in length. A ClustAGE plot in Fig. 4e represented the distribution of accessory genomes of the six strains. The characteristics of accessory genes into the pan-genome were dominated by putative N-acetylgalactosaminyl-diphosphoundecaprenol glucuronosyltransferase, TRAP-type C4-dicarboxylate transport system, Type IV prepilin peptidase TadV/CpaA, Two-component response regulator BceR, osmotic stress regulated ATP-binding protein OpuAA, phages, prophages and plasmids. Some modified transporters involved in osmoadaptation of the strain RF1H, acquired during course of time has been shown in Additional file 1 Fig. S1. Comparative circular visualization of all the six genomes depicted some gap regions in the chromosome (Fig. 5). From outermost to the center rings represents the genomes of *S*. *roseus* strain MOSEL-ME25 (ring 1); strain CCM 3516 (ring 2); strain DSM 5351 (ring 3) strain BU-1 (ring 4); strain W12 (ring 5) and strain RF1H (ring 6). In 2.6 MBp regions acquired HTH-type transcriptional regulator MurR, Osmoregulated proline transporter OpuE, Ferrous-iron efflux pump FieF and Bicyclomycin resistance protein. IS256 family transposase ISSne1, Queuine tRNA-ribosyltransferase, Sec translocon accessory complex subunit YajC, Cell shape-determining protein MreC were found in 2.0 MBp regions. In 0.8 MBp regions Taurine imports ATP-binding protein TauB, D-inositol-3- phosphate glycosyltransferase, D-inositol-3-phosphate glycosyltransferase, O-methyltransferase/MSMEI_4947 were found. Carboxylesterase gene was present in 1.0 Mbp region with stress responsive protein 26, Teichoic acid translocation permease protein TagG.

### Antibiotic susceptibility profile

Multidrug-resistant attributes were shown against Polymyxin B, Fluconazole, Streptomycin, Penicillin and Aztreonam (Additional file 1 Table S6). Further, susceptibility test predicted by ResFinder were consistent with the wet-lab results (Additional file 1 Table S7).

### Biofilm formation

*S*. *roseus* RF1H developed black colonies in CRA plates and formed visible layer on the wall and at the base of tube, strongly indicated biofilm production. The SEM image (Fig. 2b) showed bacterial community are embedded in biofilm matrix.

### Structural and functional characterization of arginine decarboxylase (ADC)

**Fig. 6:**
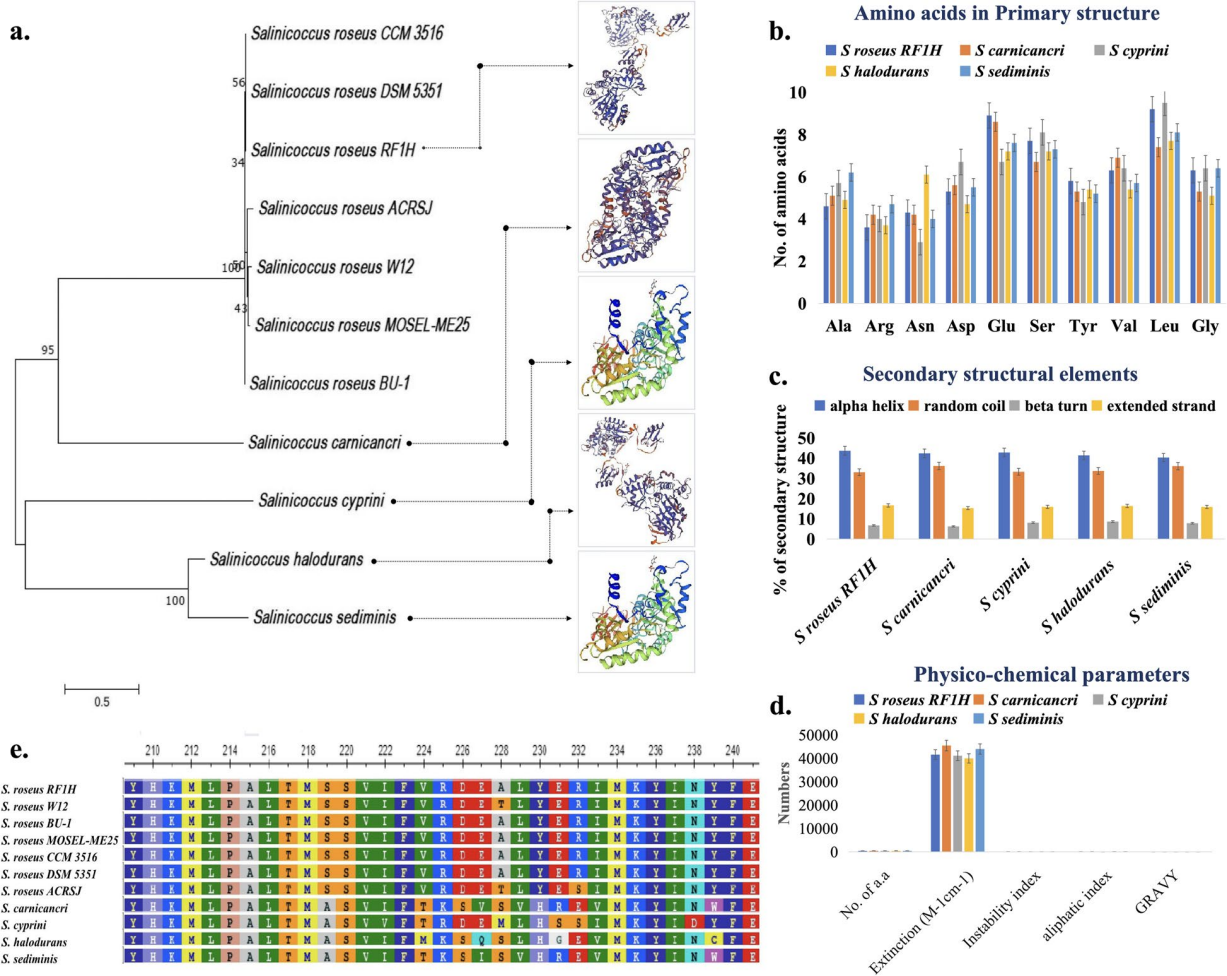
Structural and physico-biochemical features of arginine decarboxylase (ADC)- Phylogenetic tree of ADC producing *Salinicoccus* species (**a**), distribution of amino acids in the primary structure of ADC (**b**), secondary structural elements (**c**), physico-chemical characteristics of ADC (**d**), multiple sequence alignment of ADC (**e**)

From the genome annotation, we found spermidine type polyamine was responsible for the biofilm phenotype. To investigate the putative enzymes involved in biosynthesis of spermidine, we identified arginine decarboxylase is present in the genomes of *Salinicoccus* sp. The following step we carried out a detailed in silico characterization of the functional properties of ADC sequences. The phylogenetic relationship of arginine decarboxylase producing *Salinicoccus* sp. indicated that the protein was well aligned during the course of evolution. Evolutionary changes between the sequences mostly distributed into two main clades (Fig. 6a). Seven strains of* S*. *roseus* derived from the same lineage were clustered in one group with *S*. *carnicancri* from separate lineage.* S*. *roseus* strain RF1H was evolutionary close with the type strain *S*. *roseus* DSM 5351^ T^. *S*. *cyprini*, *S*. *halodurans* and *S*. *sediminis* formed another cluster. The phylogenetic tree shown the optimal tree with the sum of branch length 6.087035. Amino acid residues contributed to the primary structure of ADC protein was found highest in number in case of* S*. *carnicancri*, while *S*. *roseus* strain RF1H possessed lowest number (Fig. 6b). Polar amino acids like Asn, Glu and Ser were the most abundant amino acids presented; nonpolar amino acids like Ala, Gly, Leu, and Val were the next abundant amino acids. Presence of aspartic acid residues indicated turn-forming conformations in proteins secondary structure. The total amino acids formed helix, coils, and strands to build the secondary structure. Secondary structure predominantly made up of α-helix (~ 40%) in *S*. *roseus* RF1H and other *Salinicoccus* sp., followed by coils (~ 33%), while ~ 15% of residues were in extended strands. Lowest number of amino acids was found in (~ 6%) β-turn, as given in Fig. 6c. The model visualized in Fig. 6a depicted the proper folding of ADC protein into a three-dimensional structure. It is interesting to see that both *S*. *cyprini* and *S*. *sediminis* originated from different branch shared similar 3D structures of proteins. ADC contained mainly two large domains, N-terminal α-helix/β-barrel domain followed by hairpin turns, required pyridoxal phosphate (PLP) , and divalent cation (Mg^2+^) as cofactor for the catalytic action. The extinction coefficient (EC) of protein ranges from 39895 to 45395 M^−1^ cm^−1^ at 280 nm.

Other physico-chemical parameters such as instability index, aliphatic index, and hydropathicity index (GRAVY) were also variable for these ADC protein sequences (Fig. 6d). The instability index in all the studied sequences were greater than 40, suggested that the protein is stable. Aliphatic index conferred the thermostability of protein, thus it can be stated that RF1H is more thermostable than others. The GRAVY value indicates the solubility of protein, weather it is hydrophobic or hydrophilic. All the studied proteins have GRAVY value less than 0. In Fig. 6e COBALT Multiple sequence alignment detected a stretch of evolutionary conserved region present uniformly at amino acid residue 75–87 “SSSSSSKVGVA” and 235–247 “TITITTIKVQR”. PLP binding motifs in ADC, “EPVDG” at amino acid 314, “SSM” at 155 were found to play the catalytic role. Three consensus motifs were uniformly distributed in all the *Salinicoccus* sequences. The best possible match amino acids sequences with a width length 50 was given in Additional file 1 Table S8. The motifs with the highest E-value (5.7e-359) consists of the amino acids, VLTDEAHGAHLDIAESFPSSSMKFASDIAIQSYHKMLPALTMSSVIFVRD. When these signature sequence motifs were compared with NCBI based CDD, they resembled to aspartate aminotransferase (AAT) superfamily with C-terminal Orn/ Lys/Ard decarboxylase domain. ADC belongs to D-amino acid subfamily (fold type IV) of the AAT protein domain family.

### Biochemical properties

A wide range of carbohydrate utilization ability was observed like ribose, fructose, starch and triple sugar by the bacterium. The formation of hollow zones by the hydrolysis of tributyrin indicated the formation of extracellular esterase. In addition, proteolytic activity of the isolate was observed. The presence of catalase may be related to a high concentration of H_2_O_2_ in the hypersaline environment. However, no positive response was found in amylase, cellulase and urease.

## Discussion

In this work, moderately halophilic bacterium *Salinicoccus roseus* RF1H showed salt tolerance capability underlying genomic framework. The isolate showed it is a good esterases (3.5 g/L) [8], protease and catalase producer, abled to grow in all the tested sugars including fructose, ribose, starch, triple sugar (lactose, sucrose, glucose) except mannose (results not shown). Analysis of the genome showed the sugar transport as well as sugar phosphorylation took place by phosphoenolpyruvate (PEP): sugar phosphotransferase system (PTS). Biofilm formation by *S*. *roseus* strain RF1H observed under SEM, is no doubt a remarkable salinity resistance phenomenon. Linking biofilm phenotypes and parallelly genome analysis explored that biofilm production regulated by quorum sensing system. The ionic interplay during QS made the exopolysacchide matrix an electro-active conductor. Thus, it offers feasibility of using biofilm in microbial electrochemical systems (MES) [19]. The six genomes of *S*. *roseus* compared here, among them strain RF1H has larger genome size and higher number of CDS. Merhej et al. suggested that variation in genome size depended on environmental complexity and lifestyle of the bacteria. For example, host-dependent bacteria have small genomes compared to free-living bacteria [20]. Genome size played crucial role in evolutionary outcome of population. The genome architecture of bacteria typically consists of small genome size and tightly packed protein-coding genes. The non-functional sequences are gradually diminished unlike eukaryotes, due to selection pressure [21]. However, *S. roseus* strain RF1H do not follow this trend. Long distance horizontal gene transfer (HGT) may correspond with larger genome size in RF1H [22]. In most of the mesophilic bacteria genetic drift and mutational bias leads to elimination of non-essential regions [23]. But in extremophiles such halophiles, non-coding parts act as buffer in response to survive nutrient-limited hostile environment. Puerta-Fernandez et al. reported the existence of small non-coding RNAs in *Bacillus halodurans*, that contributed to biochemical processes [24]. Presence of one prophage [PHAGE_Bacill_250_NC_029024(8)] in the genome of RF1H may contribute to bacterial fitness, transfer of beneficial genes. Selle et al. reported, in many cases prophages can lie in the genome for a very long time and significantly influence genome remodeling during course of evolution [25]. Therefore, it contributes important biological properties for the phage-based genetic engineering. Biosynthesis of D-cysteine specific desulfhydrase, as a powerful antibacterial agent for the growth of *Escherichia coli*. Polymyxin B and β-lactamase group of antibiotic resistance achieved by structural modification of lipid A. Such modifications resulted in resistance to cationic antimicrobial peptides (AMPs) of the innate immune system and next-generation antibiotics. These antibiotics already showed resistance to *S*. *roseus* strain RF1H when tested in MHA media. The genes for enzymes, carotenoid, metal tolerance, antibiotic resistance, hydrocarbon degradations were not located among the 17 GIs, thus suggesting the inherent potential of *S*. *roseus* strain RF1H to synthesize these molecules. This could be a consequence of functional adaptation, as previously reported [26]. Giani et al. described that the 50-carbon carotenoid, bacterioruberin, acts as water barrier and maintain membrane fluidity during salt stress [27]. Carotenoids pigment function as antioxidant and protect cells from light damage in *S*. *roseus*. Yol-D protein (in GI-2) is homologous of Lex-A in SOS response in *Staphylococcus aureus* [28]. LLM class oxidoreductase (in GI-3) is similar with *cao* cluster protein in *Streptomyces cacaoi* [29]. SDR family oxidoreductase (in GI-3) also reported in *Comamonas testosterone*, involved in the metabolism of aromatic hydrocarbons including steroids, sugars, hormones, nitrogen fixation, and antibiotic synthesis [30]. ComK protein (in GI-4) is able to uptake exogenously provided genomic DNA, a phenomenon resembles with *Bacillus subtilis* [31].

Genome composition (core and accessory components of the pangenome) of *S*. *roseus* strain RF1H reflects the strain’s ability to evolve and niche adaptation. A total of 2,163,789 coding sequences (out of 2,629,783 bp) belonged to the part of core genome of *S*. *roseus* strain RF1H. The rest of the genome belonged to the accessory and unique parts. The percentage of core and accessory completely depends on the representative species considered to compare and the calculated pangenome. Here, the core genome represents 56% of the total pangenome (3,844,355 bp) of *S*. *roseus*. Core genome size varies significantly across species (214 to 4766 genes) [32]. The rest 44% belongs to the accessory genome. Therefore, we may consider it as an open pangenome with highly adaptive features [33]. The gap regions in circular genome clearly indicates evolutionary adaptation happened in genetic level in case of strain RF1H. The acquired genes in this region made RF1H unique than other strains. Khan and Patra showed that the evolutionary adaptation to extreme saline environment attributed the preference of GAC codon (more G/C-ending codon) than non-halophiles [34]. Arella et al. suggested that co-evolution of codon bias and abundant tRNA availability are associated with adaptation of microorganisms harboring in extreme environments [35]. Because of this unique feature *S*. *roseus* strain RF1H could be used as model to study genomic evolution at hypersaline environment. Despite being non-motile, *S. roseus* RF1H adhered to the bottom of tube. This could be due to spontaneous formation of adhesive matrix or biofilm that promotes bacterial aggregates to survive in nutrient depletion, high salinity, and antibiotic exposed environment [36]. Evidence showed that many bacteria such as *Halomonas* sp., *Salinibacter* sp., *Bacillus* sp., inhabited in saline wetland or limestone were equally competent in biofilm formation [37, 38]. The essential role of polyamine spermidine in robust biofilm formation was observed in Gram-positive *Bacillus* sp. [15]. Similarly, *Salinicoccus hispanicus* contained spermidine as the major polyamine. Spermidine was the predominant compound in *S*. *sesuvii* [39]. However, appreciable amount of polyamine was not observed in *Salinicoccus roseus* strain ATCC 49258 [40]. RAST subsystem *viz*., 1,3-diaminopropane annotated the presence of putative enzyme, carboxynorspermidine dehydrogenase, in the* S*. *roseus* RF1H genome, which is likely that cells use spermidine as an important polyamine. We identified arginine decarboxylase from the genomes of *S*. *roseus* which could influence the biosynthesis of spermidine. The in silico study of ADC represented predominance of polar amino acids that contributed the hydrophilicity of protein. The negative score of GRAVY indicated that the ADC protein could be globular in nature. Also, negative GRAVY value supports that ADC is hydrophilic in nature [41]. The instability index was found greater than 40 which suggested the thermostability of protein [41]. The evolutionary relationship of arginine decarboxylase in *Salinicoccus* with different clusters revealed its genetic similarity and diversity over time. Identified conserved motifs and their discriminative amino acids properly supports this observation.

Our results, based on the available genomic information, strongly suggested that *S*. *roseus* has adopted unique strategies to survive in hypersaline environment. Moreover, the environmental factors and genetic background of strain RF1H should be further studied to improve our understanding on their functions and evolution.

## Conclusions

The present study applied a comprehensive outlook of the lifestyle, genomic features, and metabolic functionalities of *Salinicoccus roseus* strain RF1H, from the first draft whole genome analysis. The combined data of phenotypic and genome characteristics provided the information that RF1H genome might have possessed unique features to survive in hypersaline condition. The open pangenome and the accessory genome enriched with genes for defense mechanism, transportation and metabolism of organic substances are also efficient adaptation strategies of the strain in these disaster prone environment of Sundarban. The comparative genomics performed among the *S*. *roseus* strains, clearly suggested the existence of unique genetic elements that contributed to the remarkable ability of *S*. *roseus* to survive in a specific niche. The compatible solutes and biomolecules produced during metabolism may imparts useful properties in biotechnological applications. Taken altogether, the analysis of this strain unveiled positive characteristics to be considered as a good candidate for exploring lifestyle of other wetland inhabitant bacteria with future biotechnological implementations. Our results paved the way to plan further investigations at biochemical and genetic levels in *S*. *roseus*, in order to reveal a complete metabolic network of halophilic bacteria to enhance biosynthesis of metabolic products.

## Methods

### Bacterial strain, growth condition, and culture maintenance

The halophilic bacterium, *S*. *roseus* strain RF1H was isolated from soil sample, collected from riverfront area of Sundarban, West Bengal, India (latitude, longitude: 22°8′15″ N and 88°50′20″ E). The sampling location was georeferenced using QGIS software [version 3.20.1]. The temperature and pH of the sampling site was ± 25 °C and 7.8 respectively. The isolation was performed in a complex halophilic media (with slight modification of Schneegurt) [42], providing 15% sodium chloride (w/v) [media containing (g/L^−1^): tryptone 10, peptone 5, yeast extract 10, magnesium sulphate heptahydrate 25, potassium chloride 2, sodium citrate 3, agar 18; pH adjusted at 5.5]. Culture plates were incubated at 35 °C for seven days. The pure cultures were maintained at 4˚C in complex halophilic media.

### Microscopic study of the isolate

Phenotypic characters of the bacterial isolate RF1H were studied following standard microbiological methods. Gram staining of the isolate RF1H was performed using the standard protocol and visualized using compound microscope (Olympus GB107167). Morphology of the vegetative cell was studied from 12 h old culture and observed under Fluorescence Microscope (Leica, DM 1000) (100 × objective) by acridine orange fluorescent dye solution. For scanning electron microscopy (SEM), the log-phase culture of the bacterial cells were fixed in 0.25% glutaraldehyde (in 50 mM Na-Phosphate, pH 7.0), followed by gradual dehydration in ethanol. Gold coating was done by sputter and observed at an electron high tension (EHT) of 5 kV (Hitachi S-530). Ultrastructure of the isolate was further studied by transmission electron microscope (TEM) (JEOL; JEM 1400 plus) (100 × resolution) by following the same protocol of glutaraldehyde fixation and ethanol dehydration. Ultrathin sections were stained with 0.5% uranyl acetate for 2 min in 3% lead citrate and placed in copper grid. The accelerated voltage 100 kV was applied for visualization.

### Genome sequencing, assembly and annotation

#### Genomic DNA isolation and sequencing

The genomic DNA of RF1H was extracted using Zymo Research Fungal/bacterial Miniprep kit (D6005). The quality of the DNA was checked by gel electrophoresis (1.5% agarose gel) and the quantity of DNA was measured in NanoDrop 1000 version 3.1.1 spectrophotometer (Thermo Scientific, USA). Prior to library construction, purity of DNA was assessed by Qubit Fluorometer Fluorometer (ThermoFisher Scientific, USA). The libraries were constructed from 1 μg of genomic DNA by using the Illumina TrueSeq DNA PCR-Free HT library preparation kit (Illumina, USA). DNA fragmentation was performed using Covaris AFA (Covaris, USA), followed by end-repair and adapter ligation. The final libraries were denatured and sequenced using Illumina MiSeq platform (Illumina, USA).

#### Assembly and annotation

The obtained paired end reads were trimmed using Trimmomatic v.0.39 [43] and quality of the reads were determined by FASTQC v.0.11.5 tool [44]. Reads were assembled into contigs with CLC Assembly Cell v. 5.2.1 tool (accessed on 21^st^ July,2021) using the de novo assembly method. The quality of the assembly was analyzed using QUAST (v.5.0.2) tool [45]. That draft assembly of RF1H was used for the annotation. CGView^BETA^ [46] server was used to visualize the RF1H genome. The Type (Strain) Genome Server (TYGS) was used for taxonomic identification [47]. Further, Average nucleotide identity (ANI) values were calculated among the close relatives of RF1H using JSpeciesWS [48] and heatmap was generated in R (version 4.1.1) using the "pheatmap" package [49] to understand taxonomic relatedness. Rapid Annotations using Subsystems Technology (RAST) [50] and NCBI Prokaryotic Genome Annotation Pipeline (PGAP) [51] were used for the annotation of functional genes. The SEED viewer was used to predict genes, protein functions and subsystem categories [52]. Essential enzyme encoding genes were obtained from Kyoto Encyclopedia of Genes and Genomes (KEGG) [53]. Putative prophages sequences in the genome were detected by using PHASTER web server [54]. The cluster of mobile genetic elements were detected by IslandViewer 4 [55]. IslandViewer combines IslandPick (uses sequential pair-wise alignments of the query genome against each member of a group of phylogenetically related reference genomes to identify regions in the query genome not present in any of the accessory genomes), SIGI-HMM (identifies regions of codon usage dissimilar from comparator species), and IslandPath-DIMOB (examines query sequence for the presence of flanking repeats, mobility genes, nearby tRNAs, and atypical dinucleotide sequence to identify genomic regions that were potentially acquired by HGT) algorithms [56].

#### Comparative genome analysis

Comparative analyses of all the available genomes of *Salinicoccus roseus* from NCBI GenBank database [https://www.ncbi.nlm.nih.gov/genbank/] (as on 17^th^ May, 2022) i.e., *S*. *roseus* strain BU-1;* S*. *roseus* strain MOSEL-ME25; *S*. *roseus* strain CCM 3516;* S*. *roseus* strain W12; *S*. *roseus* strain DSM 5351 and* S*. *roseus* strain RF1H were performed. Pan-genomes were studied in order to identify the shared genes (core genome) and unique or variable genes (accessory genome) using Spine and AGEnt tool [57]. Spine was used to differentiate the core-genome from a group of *S*. *roseus* sequences. The genomes were aligned using the NUCmer function of the MUMmer software package. AGEnt program was then mapped the core gene sequences of each genome, filtering out core gene sequences from the reference sequence. Thus, identified the accessory (non-core) genes sequences in each genome. Distribution of the accessory genomic elements (AGEs) among the genome, also referred to as bins, were identified using ClustAGE [58]. Briefly, ClustAGE algorithm grouped similar AGE sequences into ‘bins’, bins were further subdivided into ‘subelements’ [59].CGViewer (Circular Genome Viewer) server was used to visually compare newly sequenced genome (RF1H) with reference genomes [46].

#### Antibiotic sensitivity

The bacterium was subjected to antibiotic susceptibility test using different antibiotic discs (HiMedia, Mumbai, India). Disc diffusion method was performed for this assay on Mueller–Hinton Agar (MHA) media and incubated at 35˚C for 24 h. The tested antibiotic includes- Polymyxin B (30 µg/disc), Fluconazole (25 µg/disc), Norfloxacin (10 µg/disc), Streptomycin (10 µg/disc), Penicillin (6 µg/disc), Aztreonam (30 µg/disc), Cefotaxime (30 µg/disc), Amoxicillin (10 µg/disc), Ceftriaxone (30 µg/disc) and Nalidixic acid (30 µg/disc). Antimicrobial susceptibility was also tested with the whole genome sequence of RF1H in ResFinder web server [60].

#### Biofilm formation assay

The qualitative assay for biofilm formation was performed in Congo Red Agar (CRA) method and tube method, described by [61]. The biofilm was visualized using a scanning electron microscope.

### Bioinformatics analyses of Arginine Decarboxylase (ADC)

Since the knowledge of biofilm formation in *Salinicoccus roseus* is rather limited, the genomes were screened for the presences of arginine decarboxylase enzyme which is one of the important enzymes of biofilm growth. We used bioinformatics approach to study details of ADC. For this, the FASTA sequences for arginine decarboxylase (ADC) of different species of *Salinicoccus* sp. were obtained from BV-BRC system [https://www.bv-brc.org/]. Phylogenetic analysis of the ADC producing *Salinicoccus* sequences was performed by the neighbour-joining clustering method with 1000 bootstrap replicates using MEGA X software [62]. The evolutionary distances were calculated using the Poisson correction method. Physico-chemical attributes, such as amino acid composition, extinction coefficient, instability index, aliphatic index and grand average of hydropathy (GRAVY) of the ADC proteins of *S*. *roseus* RF1H, *S. carnicancri*, *S*. *cyprini*, *S*. *halodurans* and *S*. *sediminis* were calculated by using Expasy ProtParam tool [https://web.expasy.org/protparam/]. Secondary structure prediction of ADC was carried out by SOPMA from the NPS server [https://npsa-pbil.ibcp.fr/cgi-bin/npsa_automat.pl?page=/NPSA/npsa_sopma.html]. *S*. *roseus* strain RF1H was selected to predict the tertiary structure of ADC. SWISS-MODEL [https://swissmodel.expasy.org/] was used to build 3D models and the model quality was assessed by ProSA-web [https://prosa.services.came.sbg.ac.at/prosa.php]. Multiple protein sequences were aligned with COBALT tool [https://www.ncbi.nlm.nih.gov/tools/cobalt/re_cobalt.cgi] to understand the amino acid differences in the structure. The conserved protein motifs were analyzed by MEME tool [https://meme-suite.org/meme/index.html]. The deduced domains were subjected to find protein family using the NCBI conserved domain database (CDD) [https://www.ncbi.nlm.nih.gov/cdd].

### Biochemical characteristics

Qualitative detection of extracellular hydrolytic enzymes like amylase, cellulase, protease, lipase/esterase, catalase and carbohydrate fermentation using various sugars like- ribose, fructose, starch, mannose and triple sugar (lactose, sucrose, glucose and iron) were performed on agar plate assays [63].

### Statistical analyses

Statistical analyses of the basic genomic features like genome length, GC content, number of CDS etc. were performed with a t-test in R Studio [47]. The *p*-value of < 0.05 was considered as significant threshold. Experiments were performed in triplicates. Standard errors were calculated and shown in charts as error bars.

### Supplementary Information

**Additional file 1: Supplementary Table S1.** Matrix consisting of Average Nucleotide Identity (ANI) values of the fourteen genomes of Salinicoccus. **Supplementary Table S2.** RAST subsystem analysis for proteins involved in various metabolic activities of Salinicoccus roseus RF1H. **Supplementary Table S3.** Functional annotation of genes present in Genomic Islands of S. roseus strain RF1H. **Supplementary Table S4.** Assembly and annotation report of all available Salinicoccus roseus genomes from NCBI GenBank. **Supplementary Table S5.** Features assigned to subsystems from RAST server present in all S. roseus strains. **Supplementary Table S6.** Antibiotic sensitivity of Salinicoccus roseus strain RF1H. **Supplementary Table S7.** ResFinder FESA server generated antimicrobial test results of S. roseus RF1H. **Supplementary Table S8.** Distribution of three motifs with best possible amino acid. **Supplementary Figure S1.** Osmoadaptation strategies of S. roseus revealed by genome analysis.

## Data Availability

The whole genome shotgun project of *Salinicoccus roseus* strain RF1H has been deposited in NCBI GenBank under the accession number JAIMFU010000000.1 (BioProject number PRJNA756885 and BioSample number SAMN20929570). Supplementary material related to this article is available online.

## Abbreviations

ABC transporter: ATP-binding cassette transporter
ADC: Arginine decarboxylases
AGEs: Accessory Genomic Elements
AI: Autoinducer
AIP: Autoinducing Peptide
AMP: Antimicrobial Peptides
ANI: Average nucleotide identity
BLAST: Basic Local Alignment Search Tool
CGViewer: Circular Genome Viewer
CRISPR: Clustered Regularly Interspaced Short Palindromic Repeats
EHT: Electron High Tension
GBDP: Genome BLAST Distance Phylogeny
GI: Genomic Island
HGT: Horizontal Gene Transfer
KEGG: Kyoto Encyclopedia of Genes and Genomes
MES: Microbial Electrochemical Systems
MHA: Mueller–Hinton Agar
NCBI: National Centre for Biotechnology Information
QS: Quorum Sensing
RAST: Rapid Annotations using Subsystems Technology
SEM: Scanning Electron Microscopy
TEM: Transmission Electron Microscope
TYGS: Type (Strain) Genome Server
