# Supplementary material for: Delving into the lifestyle of Sundarban Wetland resident, biofilm producing, halotolerant Salinicoccus roseus: a comparative genomics-based intervention
Source: BMC Genomics. 2023 Nov 13;24:681. doi: 10.1186/s12864-023-09764-w (PMC10642018; doi:10.1186/s12864-023-09764-w)
Supplement: Supplementary file 1 — Additional file 1: Supplementary Table S1. Matrix consisting of Average Nucleotide Identity (ANI) values of the fourteen genomes of Salinicoccus. Supplementary Table S2. RAST subsystem analysis for proteins involved in various metabolic activities of Salinicoccus roseus RF1H. Supplementary Table S3. Functional annotation of genes present in Genomic Islands of S. roseus strain RF1H. Supplementary Table S4. Assembly and annotation report of all available Salinicoccus roseus genomes from NCBI GenBank. Supplementary Table S5. Features assigned to subsystems from RAST server present in all S. roseus strains. Supplementary Table S6. Antibiotic sensitivity of Salinicoccus roseus strain RF1H. Supplementary Table S7. ResFinder FESA server generated antimicrobial test results of S. roseus RF1H. Supplementary Table S8. Distribution of three motifs with best possible amino acid. Supplementary Figure S1. Osmoadaptation strategies of S. roseus revealed by genome analysis. [file 12864_2023_9764_MOESM1_ESM.doc]

# **Journal Name: BMC Genomics**

# **Title page of Supplementary Material**

**Delving into the lifestyle of Sundarban Wetland resident, biofilm producing*,* halotolerant *Salinicoccus roseus*: a comparative genomics-based intervention**

Bhramar Dutta^1^, Urmi Halder^1^, Annapurna Chitikineni^2,3^, Rajeev K. Varshney^2,3^, Rajib Bandopadhyay^1*^

^1^Microbiology Section, Department of Botany, The University of Burdwan, Burdwan, West Bengal-713104, India;

^2^Center of Excellence in Genomics and Systems Biology, International Crops Research Institute for the Semi-Arid Tropics (ICRISAT), Hyderabad, India;

^3^State Agricultural Biotechnology Centre, Centre for Crop and Food Innovation, Murdoch University, Murdoch 6500, Australia

**^*^Correspondence to** Rajib Bandopadhyay

**Email** [rajibindia@gmail.com](mailto:rajibindia@gmail.com)

**Telephone** (0342) 2656427

**Fax** 91-0342-2656427

**ORCID iD of authors**

Bhramar Dutta **0000-0003-1694-0366**

Rajeev K. Varshney **0000-0002-4562-9131**

Rajib Bandopadhyay **0000-0002-8318-5631**

**Supplementary Table S1: Matrix consisting of Average Nucleotide Identity (ANI) values of the fourteen genomes of *Salinicoccus***

|  | ***S roseus* RF1H** | ***S alkaliphilus*** | ***S carnicancri*** | ***S cyprini*** | ***S halitifaciens*** | ***S halodurans*** | ***S hispanicus*** | ***S kekensis*** | ***S sediminis*** | ***S roseus* BU-1** | ***S roseus* CCM 3516** | ***S roseus* DSM 5351** | ***S roseus* MOSEL-ME25** | ***S roseus* W12** |
| --- | --- | --- | --- | --- | --- | --- | --- | --- | --- | --- | --- | --- | --- | --- |
| ***S roseus* RF1H** | 100 | 70.16 | 72.83 | 77.48 | 70.64 | 72.76 | 77.14 | 70.47 | 73.46 | 95.75 | 95.61 | 95.68 | 96.02 | 95.46 |
| ***S alkaliphilus*** | 70.21 | 100 | 69.69 | 69.71 | 82.18 | 69.71 | 69.31 | 82.07 | 69.95 | 70.13 | 70.04 | 70.1 | 70.02 | 70.19 |
| ***S carnicancri*** | 72.99 | 69.62 | 100 | 72.53 | 69.9 | 79.91 | 72.29 | 69.88 | 92.89 | 72.94 | 73.04 | 73.04 | 72.89 | 72.83 |
| ***S cyprini*** | 77.75 | 69.78 | 72.53 | 100 | 70.05 | 72.62 | 85.9 | 69.86 | 72.94 | 77.65 | 77.68 | 77.72 | 77.74 | 77.66 |
| ***S halitifaciens*** | 70.54 | 81.86 | 69.68 | 69.88 | 100 | 69.58 | 69.5 | 92.41 | 70.17 | 70.47 | 70.42 | 70.44 | 70.35 | 70.37 |
| ***S halodurans*** | 73.13 | 69.78 | 79.76 | 72.91 | 69.85 | 100 | 72.45 | 69.83 | 80.73 | 72.93 | 73.23 | 73.25 | 73.2 | 72.78 |
| ***S hispanicus*** | 77.37 | 69.28 | 72.28 | 85.9 | 69.59 | 72.48 | 100 | 69.51 | 72.43 | 77.36 | 77.3 | 77.34 | 77.38 | 77.45 |
| ***S kekensis*** | 70.29 | 82.03 | 69.71 | 69.71 | 92.71 | 69.62 | 69.41 | 100 | 70.12 | 70.32 | 70.28 | 70.31 | 70.2 | 70.27 |
| ***S sediminis*** | 73.65 | 70.04 | 92.72 | 73.1 | 70.45 | 80.92 | 72.56 | 70.39 | 100 | 73.55 | 73.52 | 73.59 | 73.78 | 73.26 |
| ***S roseus* BU-1** | 96.1 | 70.15 | 72.86 | 77.49 | 70.61 | 72.78 | 77.15 | 70.45 | 73.29 | 100 | 97.1 | 97.15 | 96.28 | 96.14 |
| ***S roseus* CCM 3516** | 96.09 | 70.15 | 72.87 | 77.52 | 70.53 | 73.04 | 77.13 | 70.36 | 73.45 | 97.04 | 100 | 99.94 | 96.26 | 95.96 |
| ***S roseus* DSM 5351** | 96.12 | 70.07 | 72.82 | 77.7 | 70.47 | 72.9 | 77.4 | 70.4 | 73.4 | 97.23 | 99.93 | 100 | 96.41 | 96.12 |
| ***S roseus* MOSEL-ME25** | 96.58 | 70.06 | 72.85 | 77.71 | 70.45 | 72.95 | 77.34 | 70.31 | 73.7 | 96.27 | 96.24 | 96.31 | 100 | 95.58 |
| ***S roseus* W12** | 95.8 | 70.17 | 72.81 | 77.59 | 70.52 | 72.74 | 77.36 | 70.45 | 73.17 | 96.26 | 96.1 | 96.13 | 95.52 | 100 |

**Supplementary Table S2: RAST subsystem analysis for proteins involved in various metabolic activities of *Salinicoccus roseus* RF1H**

| **Subsystem** | **Genes/proteins involved** | |
| --- | --- | --- |
| ***Osmoadaptation*** | | |
| Glycine betaine | Glycine N-methyltransferase (EC 2.1.1.20) | |
|  | Sarcosine N-methyltransferase |  |
|  | Dimethylglycine N-methyltransferase |  |
| ABC transporter | ABC-type Fe^3+^ transport system, periplasmic component | |
|  | ABC-type spermidine/putrescine transport systems, ATPase components |  |
|  | ABC Fe^3+^ siderophore transporter, inner membrane subunit |  |
| ATP-dependent efflux pump | ABC transporter multidrug efflux pump, fused ATP-binding domains | |
|  | Predicted membrane fusion protein (MFP) component of efflux pump, membrane anchor protein YbhG |  |
| Adaptation to d-cysteine | D-cysteine desulfhydrase (EC 4.4.1.15) | |
|  | L-Cystine ABC transporter, periplasmic cystine-binding protein |  |
| Hyperosmotic potassium uptake | Trk system potassium uptake protein TrkA | |
|  | K(+)-uptake protein KtrB, integral membrane subunit |  |
| Osmotic stress cluster | Osmotically inducible lipoprotein B precursor | |
|  | Heat shock (predicted periplasmic) protein YciM, precursor |  |
|  | Phosphatidylglycerophosphatase B (EC 3.1.3.27) |  |
| ***Morphology*** | | |
| C30 -Carotenoid | 4,4'-diapolycopene oxidases | |
|  | Squalene desaturase |  |
| C40 -Carotenoid | Hydroxyneurosporene dehydrogenase | |
|  | Spheroidene/spirilloxanthin monooxygenase |  |
| C50 -Carotenoid | Lycopene elongase (EC 2.5.1.-) | |
| Gammaproteobacteria | 4-diphosphocytidyl-2-C-methyl-D-erythritol kinase (EC 2.7.1.148) | |
|  | Ribose-phosphate pyrophosphokinase (EC 2.7.6.1) |  |
|  | Outer membrane lipoprotein LolB |  |
|  | 2-Keto-3-deoxy-D-manno-octulosonate-8-phosphate synthase (EC 2.5.1.55) |  |
| Gram positive competence | Late competence protein ComEA, DNA receptor | |
|  | ComF operon protein A, DNA transporter ATPase |  |
|  | Late competence protein ComC, processing protease |  |
| Flagellar motility | Flagellar motor rotation protein MotA, Mot B | |
|  | Flagellar motor switch protein FliM | |
| ***Environmental stress*** | | |
| Cold shock protein | CspA, CspB, CspC, CspD, CspE, CspF | |
| Heat shock protein | DNA polymerase III delta prime subunit (EC 2.7.7.7) | |
|  | Heat shock protein HtrA |  |
|  | Mycobacterial persistence regulator MprA |  |
|  | UTP--glucose-1-phosphate uridylyltransferase (EC 2.7.7.9) |  |
| Quorum Sensing | Autoinducer 2 (AI-2) kinase LsrK (EC 2.7.1.-) | |
|  | Autoinducer 2 (AI-2) ABC transport system, periplasmic AI-2 binding protein LsrB |  |
|  | LsrR, transcriptional repressor of lsr operon |  |
|  | Autoinducer 2 sensor kinase/phosphatase LuxQ  (EC 2.7.3.-) (EC 3.1.3.-) |  |
| ***Hydrocarbon degradation*** | | |
| Chloroaromatic degradation | 3-Oxoadipate enol-lactonase (EC 3.1.1.24) | |
|  | Beta-ketoadipyl CoA thiolase (EC 2.3.1.-) |  |
|  | Chlorocatechol 1,2-dioxygenase |  |
| Anaerobic biodegradation of toluene and ethylbenzene | Benzoylsuccinyl-CoA thiolase alpha subunit (EC:2.3.1.-) | |
|  | Phenylitaconyl-CoA hydratase (EC 4.2.1.-) | |
|  | Benzoylacetate CoA-ligase |  |
|  | 2-[hydroxy(phenyl)methyl]-succinyl-CoA dehydrogenase alpha subunit (EC 1.1.1.35) |  |
| Biphenyl Degradation | Biphenyl-2,3-diol 1,2-dioxygenase (EC 1.13.11.39) | |
|  | 2-keto-4-pentenoate hydratase (EC 4.2.1.80) |  |
|  | 2,3-dihydroxy-4-phenylhexa-4,6-diene dehydrogenase |  |
| ***Antibiotic resistance*** | | |
| Beta-lactamase resistant | Beta-lactamase class C and other penicillin binding proteins  Beta-lactamase AmpS | |
|  | Beta-lactamase (Cephalosporinase) (EC 3.5.2.6) |  |
| Polymyxin resistance | Polymyxin resistance protein PmrL, sucrose-6 phosphate hydrolase | |
|  | Polymyxin resistance protein ArnC, glycosyl transferase |  |

**Supplementary Table S3:** **Functional annotation of genes present in Genomic Islands of *S*. *roseus* strain RF1H**

| **Genomic Island** | **Island end (bp)** | **Product External Annotations** | | |
| --- | --- | --- | --- | --- |
| **GI-1** | 24099 | CpsD/CapB family tyrosine-protein kinase | | |
|  |  | polysaccharide biosynthesis protein | | |
|  |  | DegT/DnrJ/EryC1/StrS family aminotransferase | | |
|  |  | sugar transferase | |  |
|  |  | GNAT family N-acetyltransferase | | |
|  |  | glycosyltransferase family 1 protein | | |
|  |  | glycosyltransferase family 4 protein | | |
|  |  | capsular polysaccharide biosynthesis protein CapF | | |
|  |  | UDP-N-acetylglucosamine 2-epimerase (non-hydrolyzing) | | |
|  |  | CatB-related O-acetyltransferase | | |
|  |  | O-antigen ligase family protein | | |
|  |  | polysaccharide pyruvyl transferase family protein | | |
|  |  | murein biosynthesis integral membrane protein MurJ | | |
|  |  | nitroreductase family protein | | |
|  |  | UTP--glucose-1-phosphate uridylyltransferase GalU | | |
|  |  | transposase family protein | | |
|  |  | capsule biosynthesis protein | | |
|  |  |  |  |  |
| **GI-2** | 20044 | phage minor capsid protein | | |
|  |  | capsid protein | |  |
|  |  | bacteriophage Gp15 family protein | | |
|  |  | phage tail tape measure protein | | |
|  |  | phage tail family protein | | |
|  |  | phage tail protein | |  |
|  |  | hemolysin XhlA family protein | | |
|  |  | holin |  |  |
|  |  | LysM peptidoglycan-binding domain-containing protein | | |
|  |  | YolD-like family protein | | |
|  |  |  |  |  |
| **GI-3** | 24807 | DUF896 domain-containing protein | | |
|  |  | LLM class flavin-dependent oxidoreductase | | |
|  |  | SprT-like domain-containing protein | | |
|  |  | MurR/RpiR family transcriptional regulator | | |
|  |  | sodium/solute symporter | | |
|  |  | SDR family oxidoreductase | | |
|  |  | M20 family metallopeptidase | | |
|  |  | M28 family peptidase | | |
|  |  | NADPH dehydrogenase | | |
|  |  | 2-dehydropantoate 2-reductase | | |
|  |  | SprT-like domain-containing protein | | |
|  |  | retron Ec67 family RNA-directed DNA polymerase/endonuclease | | |
|  |  | transposase | |  |
|  |  | site-specific DNA-methyltransferase | | |
|  |  | DNA cytosine methyltransferase | | |
|  |  | AAA family ATPase | | |
|  |  | ABC transporter ATP-binding protein/permease | | |
|  |  |  |  |  |
| **GI-4** | 8845 | thermonuclease family protein | | |
|  |  | sodium:proton antiporter | | |
|  |  | IS30 family transposase | | |
|  |  | competence protein ComK | | |
|  |  | IDEAL domain-containing protein | | |
|  |  |  |  |  |
| **GI-5** | 19776 | 50S ribosomal protein L33 | | |
|  |  | zinc ABC transporter substrate-binding protein | | |
|  |  | secondary thiamine-phosphate synthase enzyme YjbQ | | |
|  |  | HNH endonuclease | |  |
|  |  | recombinase family protein | | |
|  |  | helix-turn-helix domain-containing protein | | |
|  |  | recombinase family protein | | |
|  |  | DUF2188 domain-containing protein | | |
|  |  | NAD/NADP octopine/nopaline dehydrogenase family protein | | |
|  |  | (deoxy)nucleoside triphosphate pyrophosphohydrolase | | |
|  |  | helix-turn-helix domain-containing protein | | |
|  |  |  |  |  |
| **GI-6** | 6909 | secondary thiamine-phosphate synthase enzyme YjbQ | | |
|  |  | HNH endonuclease | |  |
|  |  |  |  |  |
| **GI-7** | 4488 | recombinase family protein | | |
|  |  | helix-turn-helix domain-containing protein | | |
|  |  | DUF2188 domain-containing protein | | |
|  |  |  |  |  |
| **GI-8** | 4166 | type I glyceraldehyde-3-phosphate dehydrogenase | | |
|  |  | kinase/pyrophosphorylase | | |
|  |  |  |  |  |
| **GI-9** | 8701 | molybdate ABC transporter substrate-binding protein | | |
|  |  | NADP-dependent phosphogluconate dehydrogenase | | |
|  |  | preprotein translocase subunit SecY | | |
|  |  |  |  |  |
| **GI-10** | 5807 | metalloregulator ArsR/SmtB family transcription factor | | |
|  |  | molybdenum cofactor biosynthesis protein | | |
|  |  | sodium:proton antiporter | | |
|  |  | ZIP family metal transporter | | |
|  |  |  |  |  |
| **GI-11** | 4963 | cation:proton antiporter | | |
|  |  | arginase family protein | | |
|  |  |  |  |  |
| **GI-12** | 6761 | iron chaperone | |  |
|  |  | hemolysin III family protein | | |
|  |  | transcription termination/antitermination protein NusA | | |
|  |  |  |  |  |
| **GI-13** | 4039 | heavy metal translocating P-type ATPase | | |
|  |  | DUF4256 domain-containing protein | | |
|  |  |  |  |  |
| **GI-14** | 6624 | Gfo/Idh/MocA family oxidoreductase | | |
|  |  | TetR/AcrR family transcriptional regulator | | |
|  |  | formate acetyltransferase | | |
|  |  | serine hydroxymethyltransferase | | |
|  |  |  |  |  |
| **GI-15** | 12609 | peptidylprolyl isomerase | | |
|  |  | DUF2382 domain-containing protein | | |
|  |  | 30S ribosomal protein S18 | | |
|  |  | ferrous iron transport protein A | | |
|  |  | glutamyl-tRNA reductase | | |
|  |  | PAS domain-containing sensor histidine kinase | | |
|  |  | arginase |  |  |
|  |  | methyltransferase domain-containing protein | | |
|  |  | bifunctional hydroxymethylpyrimidine kinase/phosphomethylpyrimidine kinase | | |
|  |  | toxic anion resistance protein | | |
|  |  | FCD domain-containing protein | | |
|  |  |  |  |  |
| **GI-16** | 4198 | malate:quinone oxidoreductase | | |
|  |  | ATP synthase F1 subunit epsilon | | |
|  |  | anion permease | |  |
|  |  |  |  |  |
| **GI-17** | 5280 | FAD-dependent oxidoreductase | | |
|  |  | Hsp70 family protein | | |
|  |  | PLP-dependent transferase | | |
|  |  | PLP-dependent transferase | | |

**Supplementary Table S4: Assembly and annotation report of all available *Salinicoccus roseus* genomes from NCBI GenBank**

| **Strains** | **Isolation source** | **Size (Mb)** | **GC%** | **Scaffolds/**  **Contigs** | **CDS** | **Genes** | **tRNA** | **rRNA** | **Other**  **RNA** | **Pseudo**  **Gene** |
| --- | --- | --- | --- | --- | --- | --- | --- | --- | --- | --- |
| **RF1H** | Mangrove soil sediment from river, Sundarban, West Bengal, India; 2018 | 2.62 | 49.6 | 741 | 2,736 | 3088 | 63 | 22 | 4 | 25 |
| **W12**  *GCA_000819905.1* | Soda lake with high salinity; Inner Mongolia, China; 2013 | 2.56 | 50.00 | 14 | 2,544 | 2,619 | 58 | 2 | 4 | 11 |
| **DSM 5351^T^**  *GCA_003814515.1* | - | 2.49 | 49.50 | 6 | 2,439 | 2,533 | 59 | 7 | 4 | 24 |
| **CCM 3516**  *GCA_014634945.1* | - | 2.49 | 49.50 | 5 | 2,413 | 2,517 | 40 | 4 | 4 | 56 |
| **MOSEL-ME25**  *GCA_013359465.2* | Marine sediment; Arabian Sea, Karachi, Pakistan; 2015 | 2.68 | 48.90 | 554 | 2,624 | 2,763 | 66 | 22 | 4 | 47 |
| **BU-1**  *GCA_002265785.1* | Red colored salt from deposit; Tarija, Bolivia; 2012 | 2.54 | 49.60 | 16 | 2,515 | 2,612 | 60 | 15 | 4 | 18 |

Where “-” indicates not determined

**Supplementary Table S5: Features assigned to subsystems from RAST server present in all *S*. *roseus* strains**

**Complete genome annotation**

|  | **RF1H** | **W12** | **DSM 5351** | **CCM 3516** | **MOSEL-ME25** | **BU-1** |
| --- | --- | --- | --- | --- | --- | --- |
| **Cofactors, Vitamins, Prosthetic Groups, Pigments**  **Cell Wall and Capsule**  **Virulence, Disease and Defense**  **Potassium metabolism**  **Miscellaneous**  **Phages, Prophages, Transposable elements, Plasmids**  **Membrane Transport**  **Iron acquisition and metabolism**  **RNA Metabolism**  **Nucleosides and Nucleotides**  **Protein Metabolism**  **Cell Division and Cell Cycle**  **Regulation and Cell signaling**  **Secondary Metabolism**  **DNA Metabolism**  **Fatty Acids, Lipids, and Isoprenoids**  **Nitrogen Metabolism**  **Dormancy and Sporulation**  **Respiration**  **Stress Response**  **Metabolism of Aromatic Compounds**  **Amino Acids and Derivatives**  **Sulfur Metabolism**  **Phosphorus Metabolism**  **Carbohydrates** | 138 | 120 | 102 | 103 | 111 | 105 |
|  | 35 | 41 | 35 | 35 | 37 | 36 |
|  | 49 | 33 | 46 | 46 | 44 | 36 |
|  | 6 | 4 | 3 | 3 | 3 | 2 |
|  | 25 | 17 | 12 | 12 | 11 | 13 |
|  | 6 | 12 | 0 | 0 | 0 | 3 |
|  | 48 | 37 | 30 | 30 | 34 | 38 |
|  | 1 | 1 | 12 | 12 | 1 | 1 |
|  | 52 | 40 | 41 | 41 | 43 | 39 |
|  | 94 | 79 | 77 | 77 | 82 | 78 |
|  | 111 | 87 | 94 | 75 | 104 | 94 |
|  | 5 | 5 | 5 | 5 | 5 | 5 |
|  | 14 | 14 | 14 | 14 | 14 | 14 |
|  | 4 | 4 | 4 | 4 | 4 | 4 |
|  | 61 | 55 | 53 | 54 | 53 | 47 |
|  | 30 | 42 | 30 | 31 | 32 | 29 |
|  | 28 | 17 | 17 | 17 | 17 | 17 |
|  | 10 | 7 | 8 | 8 | 7 | 7 |
|  | 39 | 32 | 33 | 33 | 33 | 32 |
|  | 34 | 33 | 32 | 32 | 33 | 34 |
|  | 17 | 28 | 10 | 10 | 8 | 11 |
|  | 317 | 258 | 234 | 237 | 253 | 257 |
|  | 8 | 9 | 9 | 9 | 11 | 11 |
|  | 24 | 12 | 20 | 20 | 18 | 12 |
|  | 215 | 163 | 158 | 159 | 157 | 156 |

Core genome annotation

|  | **RF1H** | **W12** | **DSM 5351** | **CCM 3516** | **MOSEL-ME25** | **BU-1** |
| --- | --- | --- | --- | --- | --- | --- |
| **Cofactors, Vitamins, Prosthetic Groups, Pigments**  **Cell Wall and Capsule**  **Virulence, Disease and Defense**  **Potassium metabolism**  **Miscellaneous**  **Phages, Prophages, Transposable elements, Plasmids**  **Membrane Transport**  **Iron acquisition and metabolism**  **RNA Metabolism**  **Nucleosides and Nucleotides**  **Protein Metabolism**  **Cell Division and Cell Cycle**  **Regulation and Cell signaling**  **Secondary Metabolism**  **DNA Metabolism**  **Fatty Acids, Lipids, and Isoprenoids**  **Nitrogen Metabolism**  **Dormancy and Sporulation**  **Respiration**  **Stress Response**  **Metabolism of Aromatic Compounds**  **Amino Acids and Derivatives**  **Sulfur Metabolism**  **Phosphorus Metabolism**  **Carbohydrates** | 129 | 100 | 101 | 102 | 102 | 101 |
|  | 30 | 22 | 27 | 27 | 21 | 27 |
|  | 41 | 27 | 26 | 26 | 28 | 25 |
|  | 4 | 2 | 2 | 2 | 2 | 2 |
|  | 17 | 11 | 11 | 11 | 10 | 11 |
|  | 0 | 0 | 0 | 0 | 0 | 0 |
|  | 29 | 23 | 24 | 24 | 22 | 23 |
|  | 1 | 1 | 10 | 10 | 1 | 1 |
|  | 49 | 38 | 39 | 39 | 39 | 38 |
|  | 89 | 74 | 74 | 74 | 75 | 74 |
|  | 93 | 71 | 71 | 68 | 72 | 72 |
|  | 5 | 5 | 5 | 5 | 5 | 5 |
|  | 13 | 14 | 14 | 14 | 14 | 14 |
|  | 4 | 4 | 4 | 4 | 4 | 4 |
|  | 60 | 48 | 46 | 47 | 43 | 45 |
|  | 30 | 31 | 31 | 32 | 31 | 30 |
|  | 26 | 17 | 16 | 16 | 16 | 17 |
|  | 10 | 7 | 8 | 8 | 7 | 7 |
|  | 37 | 32 | 32 | 32 | 31 | 32 |
|  | 33 | 31 | 30 | 30 | 31 | 31 |
|  | 8 | 8 | 8 | 8 | 8 | 6 |
|  | 285 | 226 | 224 | 225 | 232 | 228 |
|  | 8 | 5 | 5 | 5 | 5 | 5 |
|  | 12 | 12 | 12 | 12 | 12 | 12 |
|  | 178 | 135 | 136 | 137 | 140 | 136 |

Accessory genome annotation

|  | **RF1H** | **W12** | **DSM 5351** | **CCM 3516** | **MOSEL-ME25** | **BU-1** |
| --- | --- | --- | --- | --- | --- | --- |
| **Cofactors, Vitamins, Prosthetic Groups, Pigments**  **Cell Wall and Capsule**  **Virulence, Disease and Defense**  **Potassium metabolism**  **Miscellaneous**  **Phages, Prophages, Transposable elements, Plasmids**  **Membrane Transport**  **Iron acquisition and metabolism**  **RNA Metabolism**  **Nucleosides and Nucleotides**  **Protein Metabolism**  **Cell Division and Cell Cycle**  **Regulation and Cell signaling**  **Secondary Metabolism**  **DNA Metabolism**  **Fatty Acids, Lipids, and Isoprenoids**  **Nitrogen Metabolism**  **Dormancy and Sporulation**  **Respiration**  **Stress Response**  **Metabolism of Aromatic Compounds**  **Amino Acids and Derivatives**  **Sulfur Metabolism**  **Phosphorus Metabolism**  **Carbohydrates** | 6 | 16 | 0 | 0 | 2 | 4 |
|  | 7 | 15 | 4 | 4 | 11 | 9 |
|  | 7 | 5 | 20 | 20 | 19 | 9 |
|  | 0 | 0 | 0 | 0 | 0 | 0 |
|  | 8 | 6 | 1 | 1 | 1 | 2 |
|  | 5 | 11 | 0 | 0 | 0 | 4 |
|  | 10 | 13 | 5 | 5 | 4 | 7 |
|  | 0 | 0 | 0 | 0 | 0 | 0 |
|  | 1 | 0 | 0 | 0 | 0 | 0 |
|  | 1 | 2 | 0 | 0 | 3 | 0 |
|  | 2 | 1 | 6 | 6 | 11 | 5 |
|  | 0 | 0 | 0 | 0 | 0 | 0 |
|  | 1 | 0 | 0 | 0 | 0 | 0 |
|  | 0 | 0 | 0 | 0 | 0 | 0 |
|  | 0 | 1 | 5 | 5 | 7 | 0 |
|  | 1 | 2 | 0 | 0 | 2 | 0 |
|  | 0 | 0 | 0 | 0 | 0 | 0 |
|  | 0 | 0 | 0 | 0 | 0 | 0 |
|  | 0 | 0 | 1 | 1 | 1 | 0 |
|  | 0 | 2 | 2 | 2 | 2 | 3 |
|  | 5 | 20 | 2 | 2 | 0 | 5 |
|  | 12 | 10 | 3 | 3 | 8 | 17 |
|  | 0 | 4 | 5 | 5 | 6 | 6 |
|  | 0 | 0 | 0 | 0 | 0 | 0 |
|  | 20 | 11 | 20 | 20 | 3 | 9 |

**Supplementary Table S6: Antibiotic sensitivity of *Salinicoccus roseus* strain RF1H**

| **Antibacterial agents** | **Polymyxin B**  **(30 mcg)** | **Fluconazole**  **(25 mcg)** | **Norfloxacin**  **(10 mcg)** | **Cefotaxime**  **(30/10 mcg)** | **Streptomycin**  **(10 mcg)** | **Penicillin**  **(6 mcg)** | **Amoxicillin**  **(10 mcg)** | **Ceftriaxone (30 mcg)** | **Aztreonam (30 mcg)** | **Nalidixic acid (30 mcg)** |
| --- | --- | --- | --- | --- | --- | --- | --- | --- | --- | --- |
| Zone of inhibition (mm) | R | R | 23 | 30 | R | R | 30 | 18 | R | 43 |

Where “R” denotes resistant

**Supplementary Table S7:** **ResFinder FESA server generated antimicrobial test results of *S*. *roseus* RF1H**

**
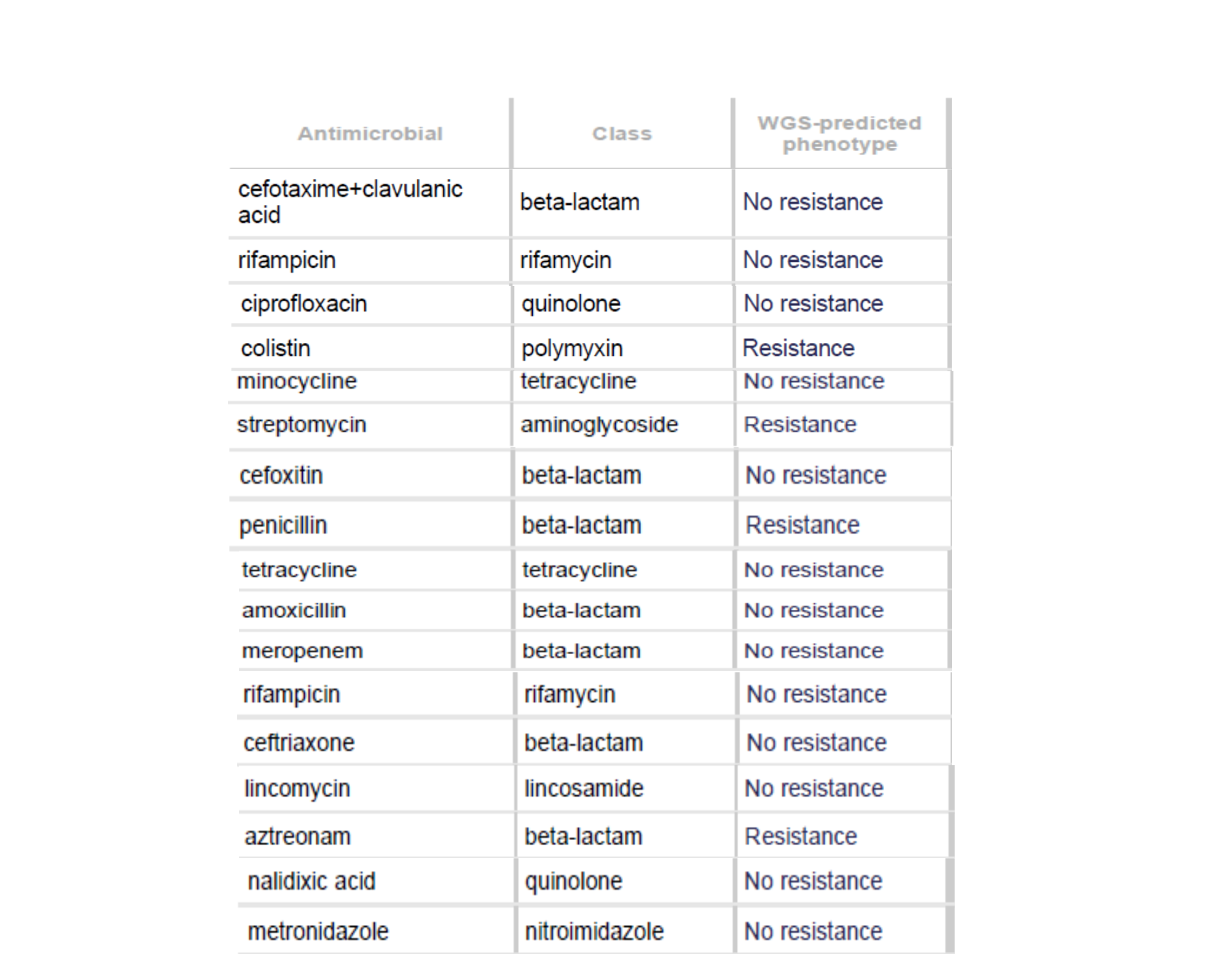
**

**Supplementary Table S8: Distribution of three motifs with best possible amino acid**

| **Motif** | **Width** | **E-value** | **Best possible amino acid** |
| --- | --- | --- | --- |
| 1 | 50 | 5.7e-359 | VLTDEAHGAHLDIAESFPSSSMKFASDIAIQSYHKMLPALTMSSVIFVRD |
| 2 | 50 | 5.5e-348 | LDWSYDMTEIEGLDDLHDPGEVLSRLNVNJAEKYEGYKAQLMVNGTTNGI |
| 3 | 50 | 2.7e-332 | CVKSIVPYPPGVPLVHENEVITGSHLKSIKHYLHNHVRIEGIKYNJQYYN |

**Supplementary Figure S1: Osmoadaptation strategies of *S*. *roseus* revealed by genome analysis**
